# Linking Isotopes and Panmixia: High Within-Colony Variation in Feather δ2H, δ13C, and δ15N across the Range of the American White Pelican

**DOI:** 10.1371/journal.pone.0150810

**Published:** 2016-03-14

**Authors:** Matthew W. Reudink, Christopher J. Kyle, Ann E. McKellar, Christopher M. Somers, Robyn L. F. Reudink, T. Kurt Kyser, Samantha E. Franks, Joseph J. Nocera

**Affiliations:** 1 Forensic Science Department, Trent University, Peterborough, Ontario, Canada; 2 Environment Canada, Saskatoon, Saskatchewan, Canada; 3 Department of Biology, University of Regina, Regina, Saskatchewan, Canada; 4 British Columbia Ministry of Forests, Lands, and Natural Resource Operations, Kamloops, British Columbia, Canada; 5 Department of Geological Sciences and Geological Engineering, Queen’s University, Kingston, Ontario, Canada; 6 British Trust for Ornithology, Thetford, Norfolk, United Kingdom; 7 Ontario Ministry of Natural Resources and Forestry, Trent University, Peterborough, Ontario, Canada; Sonoma State University, UNITED STATES

## Abstract

Complete panmixia across the entire range of a species is a relatively rare phenomenon; however, this pattern may be found in species that have limited philopatry and frequent dispersal. American white pelicans (*Pelecanus erythrorhyncos*) provide a unique opportunity to examine the role of long-distance dispersal in facilitating gene flow in a species recently reported as panmictic across its broad breeding range. This species is also undergoing a range expansion, with new colonies arising hundreds of kilometers outside previous range boundaries. In this study, we use a multiple stable isotope (*δ*^2^H, *δ*^13^C, *δ*^15^N) approach to examine feather isotopic structuring at 19 pelican colonies across North America, with the goal of establishing an isotopic basemap that could be used for assigning individuals at newly established breeding sites to source colonies. Within-colony isotopic variation was extremely high, exceeding 100‰ in *δ*^2^H within some colonies (with relatively high variation also observed for *δ*^13^C and *δ*^15^N). The high degree of within-site variation greatly limited the utility of assignment-based approaches (42% cross-validation success rate; range: 0–90% success). Furthermore, clustering algorithms identified four likely isotopic clusters; however, those clusters were generally unrelated to geographic location. Taken together, the high degree of within-site isotopic variation and lack of geographically-defined isotopic clusters preclude the establishment of an isotopic basemap for American white pelicans, but may indicate that a high incidence of long-distance dispersal is facilitating gene flow, leading to genetic panmixia.

## Introduction

Genetic panmixia, or a complete lack of genetic differentiation across the range of a species, may result when the forces that generate differentiation, such as mutation, selection and drift, are outweighed by the forces that reduce differentiation and increase homogeneity, such as gene flow. Panmixia in natural populations is rare and only expected when gene flow and dispersal are high. For example, Ward et al. [1] examined over 300 animal taxa and demonstrated that differentiation is lowest in taxa with high movement and dispersal capabilities, such as birds and flying insects. Indeed, the major commonality among species in which panmixia has been reported tends to be high movement and dispersal capabilities [2–6]. Movement and dispersal capabilities alone, however, are not necessarily good predictors of genetic panmixia [7].

Many avian taxa that have high dispersal capabilities show (sometimes strong) population differentiation, especially when gene flow is restricted by high natal philopatry and behavioural mechanisms [7,8], cryptic barriers to dispersal [9], or local adaptation [10,11]. Reudink et al. [12] and Oomen et al. [13] reported range-wide genetic panmixia in American white pelicans (*Pelecanus erythrorhyncos;* hereafter: pelicans) despite the presence of several predicted behavioural and physical barriers to gene flow. Both microsatellites [12] and mitochondrial markers [13] showed a similar lack of differentiation, suggesting high contemporary and historic patterns of gene flow, respectively. Several other lines of evidence support this idea. First, pelicans can cover hundreds of kilometers while foraging [14] and can disperse long distances. Second, pelicans have recently undergone a large-scale range expansion, including new breeding attempts hundreds of kilometers beyond recent range boundaries into northern Ontario and Nunavut, Canada [15]. Finally, breeding colonies undergo repeated local extinctions and subsequent recolonization events [16], though these events are most often due to abandonment, not mortality. These repeated colony extinctions and recolonization events may re-distribute individuals at broad spatial scales. We suggest that the recent and rapid range expansion of pelicans, e.g., at the eastern edge of their range in Canada, coupled with high levels of gene flow through long-distance dispersal, has likely resulted in an absence of genetic structure. Currently, we lack any information on patterns of breeding dispersal and the provenance of individuals establishing new colonies.

One method for tracking dispersal events is to identify the spatial distribution of genetic variation across the range of the species and assess the origin of colonizing individuals, such as through population genetic assignment tests [17–20]. Combined with complementary approaches, such as stable isotope analysis, genetic techniques can sometimes enable delineation of movement patterns (e.g., [21,22]). However, a genetic approach requires at least some genetic structuring among potential source populations to be effective (see [20,23]). Unlike genetic variation, however, variation in biogeochemical markers, such as stable isotopes, is almost entirely dependent on contemporary extrinsic factors (e.g., precipitation, diet) that can vary predictably across the landscape [24,25]. Thus, biogeochemical markers may provide valuable information on movement patterns when genetic approaches are uninformative or indicate panmixia [26]. Here, we examine patterns of range-wide isotopic structuring in the American white pelican to determine whether isotopic signatures of feathers may be informative for creating a species-specific feather isotope basemap, which could be used to examine patterns of dispersal and movement. Because biogeochemical signatures are incorporated into pelican feathers annually during their post-breeding moult, these signatures are determined by the environment at the moult location, offering potential markers for tracking movement patterns at broad spatial scales.

In this study, our aim was to: (a) examine patterns of within- and among-colony variation in three stable isotopes (*δ*^2^H, *δ* ^3^C, *δ*^15^N) and (b) examine whether isotopic variation is sufficiently patterned to be used for geographic assignment of unknown individuals to track movement patterns in dispersing pelicans.

## Methods

### Ethics statement

Because we did not handle live animals and feather collection was conducted post-breeding, we did not require an Animal Care and Use Permit. Permission to collect and retain feather samples was granted by the Crown through the Ontario Ministry of Natural Resources & Forestry.

### Sample collection

**Fig 1:**
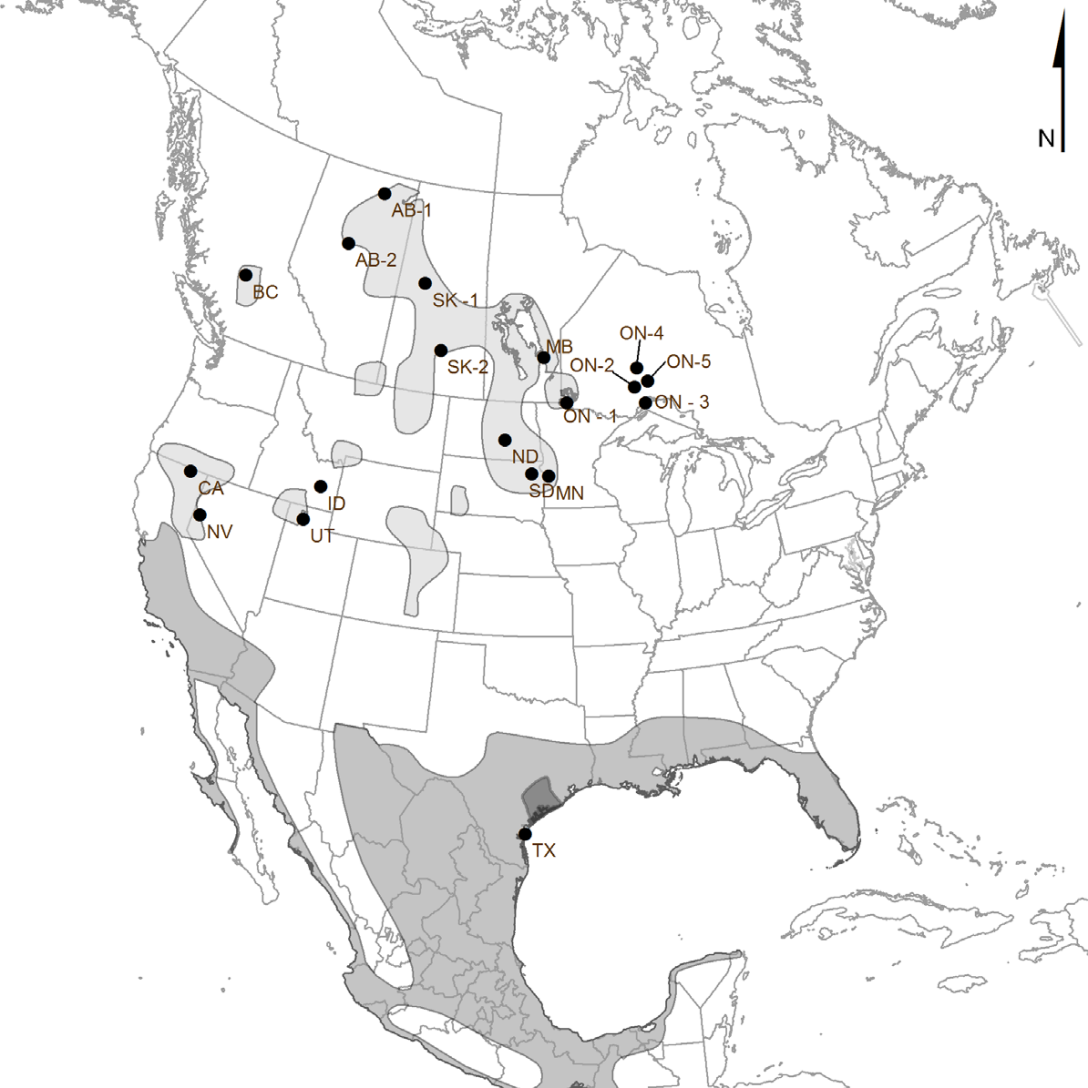
Map of Sampling Locations. Circles represent all sampled colonies; approximate main breeding range is shown in light gray, non-breeding range in medium gray, and year-round range in dark gray. Full colony names and location information available in S1 Table. The range map data were provided by NatureServe in collaboration with Robert Ridgely, James Zook, The Nature Conservancy, Conservation International, World Wildlife Fund, and Environment Canada [62].

Pelicans breed colonially, primarily in inland, freshwater lakes across the northern United States and Canada from the Pacific Coast to central Ontario/western Nunavut [14–15]. However, pelicans are prone to colony abandonment and are highly sensitive to disturbance, which generally precludes capture of adults at colonies [27,28]. To avoid such colony disturbance, we established a network of researchers across North America (including ourselves) to assist in collecting feather samples in the post-breeding period (July-August, 2008–2009) from pelican carcasses and shed, moulted feathers (*n* = 201 feathers from 19 breeding colony locations; S1 Table, Fig 1).

Definitive pre-basic moult in breeding pelicans (pelicans generally do not breed until after their fourth year), which includes the primaries and secondaries, begins during the chick-rearing stage [14, 29]. Breeding pelicans exhibit Staffelmauser (staggered moult) in primary and secondary feathers, sequentially replacing primaries, generally over two feather generations. Thus, sampled feathers (both loose feathers and those collected from carcasses) were grown either one or two years previous from the date of their collection. Because our sampled feathers were grown at the end of one of the last two previous breeding seasons, isotopic signatures in the feathers of birds returning to breed at a given colony should reflect the local environment on the breeding grounds in which that feather was grown. Although there is no evidence for it, if pelicans were to rely heavily on nutrients acquired elsewhere for reproduction (i.e., capital breeding strategy; [30]), then isotopic signatures could be altered. However, nutrients obtained by pelicans prior to arrival in March/April are unlikely to be stored long enough to be incorporated into feathers grown post-breeding.

Pelican primary feathers are easily discriminated from other pelican feathers (e.g., secondaries, remiges) and those from other species (e.g., cormorants, gulls) sharing nesting habitat. Pelicans exhibit almost entirely white plumage except on the black primaries, secondaries, and some wing coverts. Primaries are easily recognized based on size (~30–45cm; much larger than those of co-occurring species) and the amount of white edging at the feather base. One potential problem with our approach is that because we were unable to collect information on individual breeders, we risked duplicating individuals by unknowingly collecting multiple feathers from one individual. To quantify the risk of repeatedly sampling the same individual from multiple shed feathers, we genotyped a subset of individual feathers (*n* = 72) at 10 microsatellite loci and determined the likelihood of resampling the same individual based on the genetic profiles of feathers (see Methods: Microsatellite DNA analysis). We were unable to genotype all individuals, as the tissue at the base of some feathers was degraded and DNA extraction was not possible (*n*.*b*. such degradation does not affect isotopes in feathers).

### Stable isotope analyses

We analyzed the stable isotopes of three elements that occur naturally in the environment and are incorporated predictably into animal tissues. We analyzed *δ*^2^H because variation in the natural abundance of ^2^H varies with both latitude and elevation, making it particularly useful for studies of avian migration and connectivity [31]. Variation in *δ*^13^C values are associated with the proportion of C4 plants and amount of water stress experienced by plants in terrestrial biomes [32–34], but in aquatic systems high *δ*^13^C values are associated with marine-derived dietary input [35]. We also examined variation in *δ*^15^N because it increases with increasing trophic levels due to preferential incorporation of ^15^N into consumer tissues [36,37]. In terrestrial biomes, *δ*^15^N can also reflect the aridity of the environment, as it is negatively associated with rainfall and positively associated with temperature [38, 39]. Nitrogen isotope values can also be influenced by anthropogenic activity (e.g., eutrophication; [40]).

Isotopic analyses were conducted at the Queen’s Facility for Isotope Research (QFIR) in Kingston, Ontario. Feathers were washed of surface oils and debris using a 2:1 chloroform:methanol solution and air dried under a fume hood for 72 h, allowing them, and the feather standards, to equilibrate with the local atmosphere. For stable-hydrogen isotope analysis, 0.10–0.15 mg of feather barbs from the tip of pelican feathers were loaded into silver capsules and placed in an oven at 100°C for 24 h to remove any surface water, which is a necessary procedure to ensure that both exchangeable H and, in particular, absorbed water are minimized. Standard feather powder (n = 21; *δ*^2^H = -85 ‰) and two mineral standards (kaolinite *n* = 12; *δ*^2^H = -59 ‰ and brucite *n* = 8; *δ*^2^H = -96 ‰) were treated in the same way. Exchangeable H in the feathers was less than 5% of the total H and had a *δ*^2^H of -70 ‰, similar to local atmosphere, thus any correction for exchangeable H was negligible (but see [41,42]). The capsules were then crushed, loaded into a reduction furnace (Finnigan TC/EA) at 1450°C inline with an isotope ratio mass spectrometer (Finnigan MAT Delta Plus XL). Stable-hydrogen isotope ratios are reported in per mil notation (‰) relative to Vienna Standard Mean Ocean Water (V-SMOW). Measurements on the same feather were repeatable to 2 ± 2 ‰ (mean ± SD, *n* = 15), the SD of internal feather standard was 3 ‰ (*n* = 21), while SDs of repeated measures of mineral standards were both 2 ‰.

For stable-carbon and stable-nitrogen isotope analyses, samples (0.20–0.30 mg) were loaded into tin capsules, combusted in an elemental analyzer (NCS 2500), and subsequently introduced inline to an isotope-ratio mass spectrometer (Finnigan MAT 252). Stable-carbon and stable-nitrogen isotope ratios are expressed in ‰ (parts per mil) relative to the international standard V-PeeDee Belemnite for C or atmospheric N_2_ for N. The feather powder standard (n = 15; *δ*^12^C = -24.2 ‰ and *δ*^15^N = 5.7 ‰), a chicken blood standard (n = 12; *δ*^12^C = -20.7 ‰ and *δ*^15^N = 4.2 ‰), NBS-21 graphite (n = 8; *δ*^12^C = -28.1 ‰), and two NIST ammonium sulfate standards (NIST 8550, n = 4 and *δ*^15^N = -30.4 ‰; NIST 8551, n = 4 and *δ*^15^N = 53.5 ‰) were treated in the same way. Measurements on the same feather were repeatable to 0.3 ± 0.2 ‰ (*n* = 22) for carbon and 0.1 ± 0.1 ‰ (*n* = 22) for nitrogen. Repeated measurement of the standards indicate SDs of 0.2 ‰ for carbon and 0.3 ‰ for nitrogen.

### Microsatellite DNA analysis

We extracted DNA from the base of feathers using a QIAGEN DNAeasy ® Tissue Extraction kit (QIAGEN). We amplified 10 polymorphic microsatellites isolated from American white pelicans (PeEr loci; [43]) and great white pelicans (*P*. *onocrotalus*; Pel loci; [44]). For full details of amplification, visualization, and locus-specific information, see [12]. We examined allele sharing among all individuals within each sampling location to determine rates of duplication (i.e., individuals sampled more than once). Individuals were considered unique if alleles mis-matched at >1 locus by >2 base pairs.

### GIS methods

All maps and GIS analyses were completed in ArcGIS 10 (ESRI, Redlands, California, USA). We created contour maps for each isotope using ordinary kriging and the mean *δ*^2^H, *δ*^13^C, and *δ*^15^N values at the 19 feather sampling sites. Kriging is a geostatistical procedure that interpolates values based on the weighted average within a search neighborhood. We used a stable model, which minimized the mean square error and a search neighborhood of 5 with a minimum of 2. The contour maps were created to demonstrate the spatial variation in the average, or expected, feather isotope values across the species range (cf. [45]).

### Statistical analyses

Our methods for determining our ability to use multiple isotopes to assign feathers back to their original colony (cross-validation) follow [46] and [47]. Due to low sample sizes, two colonies were excluded from this analysis (Gunnison Island, UT: n = 3; Quesnel, BC: n = 1). To assign feather samples back to their most likely region of origin, we used a maximum likelihood-based assignment test [48]. We then used multivariate normal probability density functions using feather isotope values (*δ*^2^H, *δ*^13^C, *δ*^15^N) to determine the likelihood an individual originated from any given region. We used an error-incorporated resampling simulation to cross-validate known-origin feathers [49,50]. In this approach, we randomly sampled 100 values from a normal distribution based on a mean equal to the isotope value for a given individual and a standard deviation equal to the mean standard deviation for lab standards, thus producing 100 new datasets of isotope values for all individuals. We then randomly chose one of the 100 datasets to define the mean and variance-covariance of the multivariate probability density function for each region. We then determined the probability for each individual of being assigned to each region (thus producing 100 assignment outcomes per individual). This procedure was repeated 100 times, each time using a new dataset to define the mean and variance-covariance, resulting in 10,000 assignment outcomes for each individual. We classified an individual as having originated from a given region based on having the greatest number of assignments out of the 10,000 simulations. Because this approach provides a single assignment for each sample in the simulation, we could assess our confidence in our assignment (e.g., a sample that assigned 8,000 times out of 10,000 simulations had 80% confidence of assignment to that region). We opted to use a leave-one-out cross-validation approach over splitting the data in half to create training and test datasets as the latter would only be more robust with a larger dataset; the leave-one-out cross-validation approach is better suited to the small group sizes in our study (all n ≤ 20, 10/17 have n ≤ 10).

Next, to assess the potential to assign unknown origin samples, we used the resampling simulation approach described above with a leave-one-out cross validation exclusion criterion for ‘known origin’ feathers. To do this we sequentially removed each ‘known origin’ sample from the dataset and conducted an assignment procedure to determine the proportion of ‘known origin’ samples we could correctly assign to their known source. This approach broadly followed the steps above, but the individual to be assigned was excluded from the dataset when calculating the parameters (mean, variance-covariance) of the probability density function. We then examined the likelihood that an individual originated from each potential source region. We conducted all multivariate analyses using the mvnmle, mvtnorm [51,52], rrcov [53], and ade4 [54] packages for R 2.11.0 statistical software [55]. Finally, we examined the relationship between the proportion of birds correctly assigned at a colony and the range of isotopic variation at that site for each isotope using Pearson’s correlations.

To supplement the above analyses, we used k-means cluster analysis to examine whether the *δ*^2^H, *δ*^13^C, and *δ*^15^N signatures of feathers clustered into any natural groups that may be related to their geographic location. The k-means method iteratively adjusts cluster membership until the within-cluster sum-of-squares is minimized [56]. We determined the optimal number of clusters by examining plots of the within-cluster sum-of-squares versus number of clusters, ranging from two to 20. We then examined the predicted cluster assignments of feathers from each breeding colony, and we used multivariate analysis of variance (MANOVA) to determine whether there were significant differences among the selected clusters in *δ*^2^H, *δ*^13^C, and *δ*^15^N ratios. As above, Gunnison Island, UT (n = 3 feathers) and Quesnel, BC (n = 1) were excluded from the analysis. K-means clustering was conducted in R 2.11.0 [55].

## Results

### Isotopic variation

**Fig 2:**
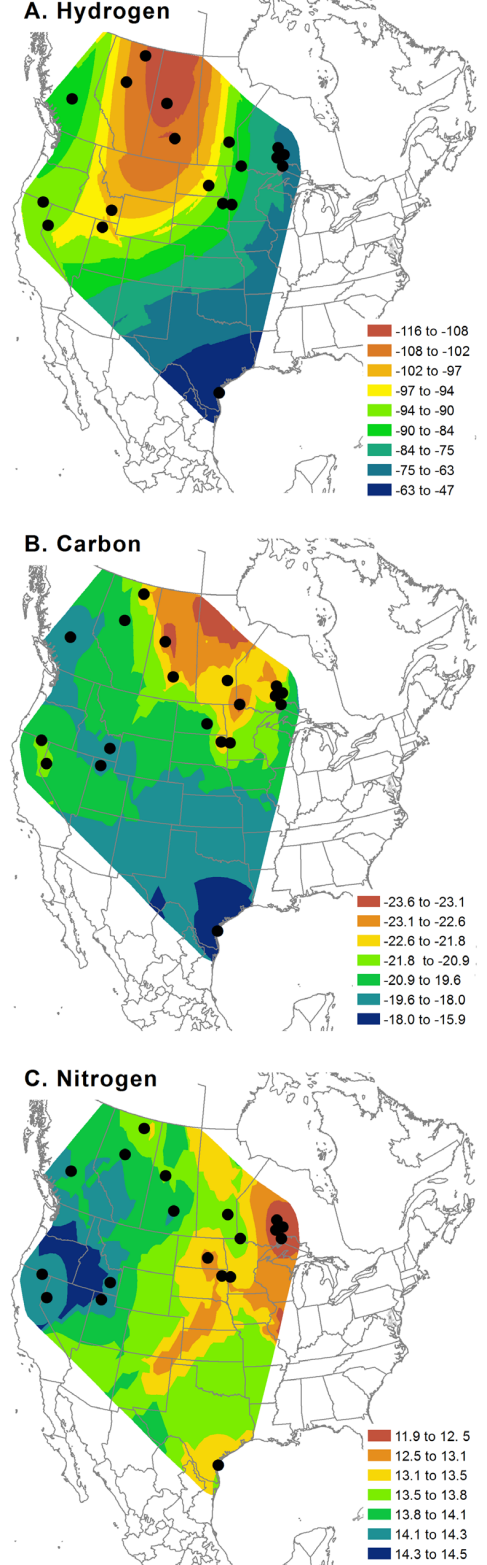
Stable Isotope Basemaps. Interpolated basemaps of isotopic variation in **A.** Hydrogen (*δ*^2^H), **B.** Carbon (*δ*^13^C), and **C.** Nitrogen (*δ*^15^N) obtained using ordinary kriging. Maps are based on the mean values of pelican feathers from 19 established American white pelican colonies.

Variation in all stable isotope ratios was high, both within and among colonies. Range-wide stable-hydrogen isotope ratios ranged from -165 to -12 ‰, while within-colony variation exceeded 100 ‰ at some colonies (S1 Table). Similar patterns were observed with stable-carbon and nitrogen isotopes, with stable-carbon isotope ratios ranging from -29.5 to -11.0 ‰ (within-colony variation < 10‰) and stable-nitrogen isotope ratios ranging from 6.7 to 19.9 ‰ (within-colony variation < 12 ‰). Interpolated isotopic basemaps of colony means of *δ*^2^H, *δ*^13^C, and *δ*^15^N illustrate patterns in the mean isotopic values across the sampling area and demonstrate the lack of clear geographic patterns (Fig 2).

### Replication of Individuals

Microsatellite DNA analysis of pelican feathers from 8 colonies indicated that 6/72 (8%) feathers matched at all loci, indicating those feathers likely originated from the same individual. Based on 10 microsatellite loci, our probability of identity (P(ID)), the probability that two individuals from a population drawn at random will share a genotype across multiple loci, was low (<0.0001). The proportion of repeated individuals varied among study locations from 0%-17% (Akimiski, NU: 0/8; Anaho Island, NV: 2/12; Boles Island, ON: 3/17; Granite Island, ON: 1/5; Lake of the Woods, ON: 0/11; Ombabika, ON: 0/5; Pipestone Rocks, MB: 0/8; San Padre Island, TX 0/6). Thus, in our analysis of 201 feathers, we estimate that ~18 might be duplicates. One of each of the identified duplicated individuals revealed by our analysis was chosen randomly for inclusion in our analyses.

### Cross-validation of samples

Our resampling approach with error incorporation revealed that individuals were correctly assigned to their source colony 42% (83/197) of the time. Of those 83 correct assignments, 59 were assigned with over 90% confidence (i.e., correctly assigned in ≥ 9,000 of the 10,000 simulations) and 82/83 assigned with >50% confidence. Assignment success rates varied across colonies, from 0% correctly assigned (Marsh Lake, MN) to 90% correct assignment (Padre Islands, TX) (Table 1, S2 Table). The proportion of birds correctly assigned was negatively correlated with the range of isotopic values at that colony for both *δ*^2^H (n = 17, r = -0.61, p = 0.01) and *δ*^13^C (n = 17, r = -0.56, p = 0.02), but not *δ*^15^N (n = 17, r = -0.26, p = 0.31).

**Table 1:** Cross-validation Success Rates.

Location	n	n correct	Proportion Correct
Anaho NWR, NV	19	3	0.16
Bitter Lake, SD	15	3	0.20
Blackfoot Reservoir, ID	9	5	0.56
Boles Island, ON	20	15	0.75
Chase Lake, ND	6	1	0.17
Clear Lake, CA	15	10	0.67
Dore Lake, SK	6	1	0.17
Granite Island, ON	8	5	0.63
Lake of the Woods, ON	10	4	0.40
Last Mountain Lake, SK	10	9	0.90
Marsh Lake, MN	15	0	0.00
Mt. St. John, ON	10	4	0.40
Ombabika Flats, ON	10	1	0.10
Padre Islands, TX	10	9	0.90
Pipestone Rocks, MB	10	5	0.50
Portage Lake, AB	15	6	0.40
Utikuma Lake, AB	9	2	0.22

Success rates of error-incorporated resampling assignment simulations using leave-one-out cross-validation. Samples were assigned to the colony of highest probability of assignment based on the results of 10,000 simulations. Locations to which each individual were assigned are reported in S2 Table.

### Cluster analysis

**Fig 3:**
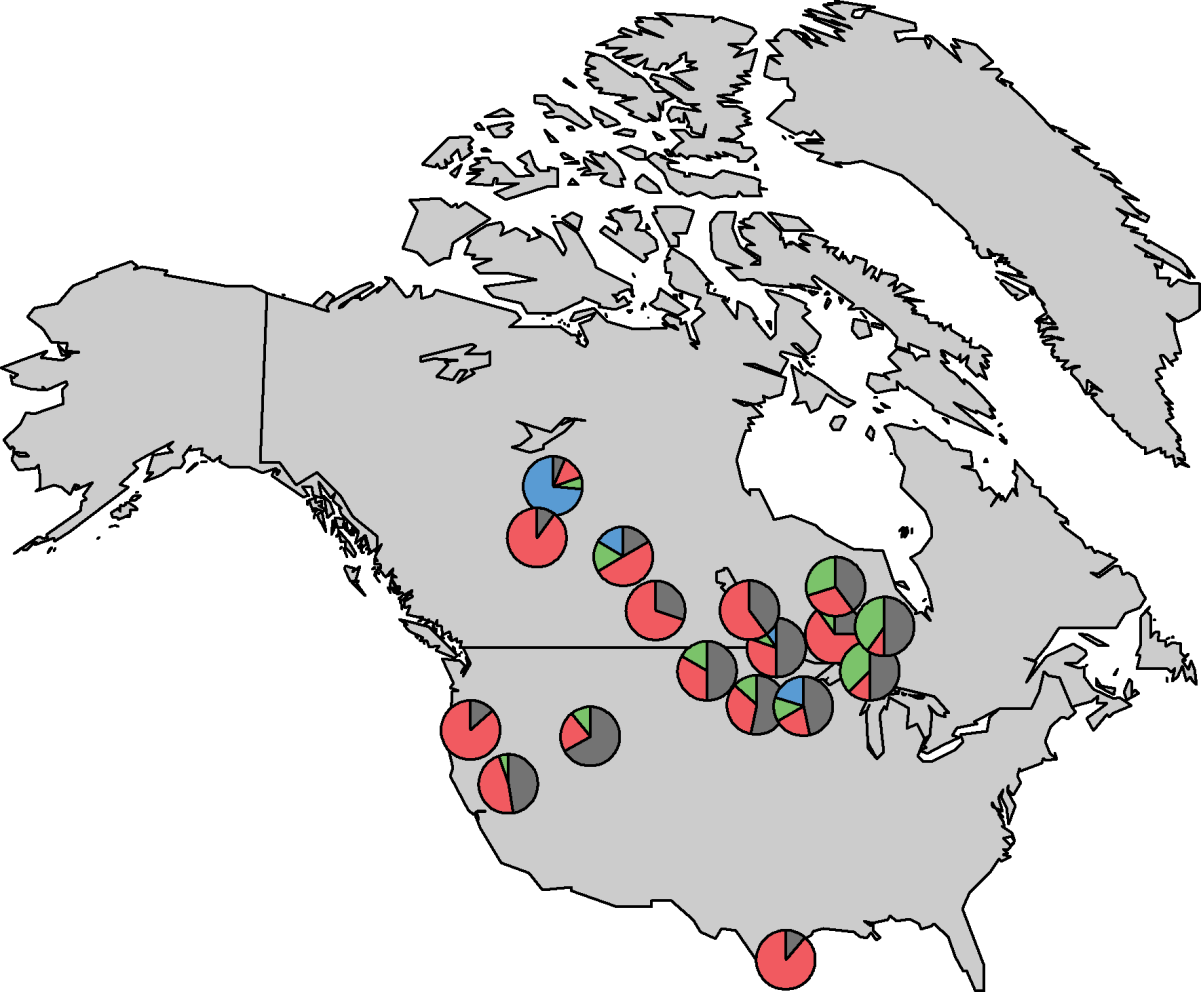
Cluster Membership Map. Proportion of feather samples from pelicans at 17 breeding colonies that fall into one of four clusters, based on k-means cluster analysis. The four colours (grey, red, blue, green) represent the proportion of samples assigned to each of the four different clusters defined by the clustering algorithm. Some colony locations have been slightly offset for better visibility.

Based on k-means clustering, the optimal number of clusters, given the data, was four (Table 2). The clusters were significantly different from one another for all three isotope ratios simultaneously (MANOVA F_3,193_ = 32.1, p < 0.001). However, cluster membership was not generally associated with geographic origin (Fig 3). In other words, clusters were spread throughout all populations, with 13 of 17 populations containing members of at least three different clusters, and no populations containing members of only one cluster.

**Table 2:** Mean (±SD) stable isotope ratios of American white pelican feathers partitioned into four clusters based on k-means clustering.

Cluster*	*δ*^2^H	*δ*^13^C	*δ*^15^N
Grey	-100.53 (7.1)	-22.73 (2.5)	13.46 (2.6)
Red	-74.74 (7.3)	-21.84 (3.5)	13.74 (2.2)
Blue	-137.83 (14.3)	-22.89 (2.7)	12.52 (2.9)
Green	-42.89 (14.1)	-18.43 (4.7)	12.34 (1.5)

* refers to colours shown in Fig 3

## Discussion

We examined the potential of stable isotopes to be used for geographic assignment of American white pelicans, a species for which genetic panmixia has recently been reported. Our results indicate a high degree of within-colony variation in stable isotopes and low cross-validation success based on colony-level assignment. Thus, we did not detect colony-level or even regional isotopic structuring, a result that mirrors the species’ lack of genetic structuring, and which we suggest may be due in part to a high incidence of breeding dispersal.

Within-colony variation was extremely high, exceeding 100 ‰ in *δ*^2^H at some colonies. Consistent with the high within-colony variation in all stable isotopes, we detected only weak geographic patterns of isotopic structuring (Fig 2). The only pelican colony that was highly distinct both in terms of absolute isotopic values and within-site variation, and also had a high proportion of correct assignments (90%) in our cross-validation was the Padre Islands, TX colony, which is situated >2200 km south of the nearest mainland colony. The Padre Islands colony is a non-migratory population along a coastal lagoon, and *δ*^2^H signatures at this site are highly enriched (-47 +/- 13 SD) and have a much smaller range than many of the mid-latitude sites (S1 Table). Similarly, the carbon isotope values of the Padre Islands samples were highly enriched (-13.4 +/- 1.4 SD) with a relatively narrow variance. This enriched *δ* ^13^C signature is consistent with a strong marine-derived dietary input, likely resulting from foraging in the hypersaline Laguna Madre [34]. Together, these results indicate that the unusual colony at Padre Islands, TX may be exclusively non-migratory rather than being composed of both non-migratory birds and northern birds staying to breed in some years. However, this possibility is tempered by the fact that our cluster analysis did not provide strong support for complete isolation of the Padre Islands colony (Fig 3).

Clustering algorithms based on *δ*^2^H, *δ*^13^C, and *δ*^15^N signatures of pelican feathers identified four distinct clusters, and yet cluster membership generally appeared unrelated to geographic origin (Fig 3). Although the Padre Islands, TX, colony appeared to be composed mostly of individuals from one cluster, members of this same cluster were found to be scattered throughout the species range. Thus, the clustering approach was similar to cross-validation in its ineffectiveness at delineating pelican colonies.

Nearly all colonies exhibited high within site isotopic variation, which was negatively correlated with success rates of cross-validation assignments. Several factors may contribute to this variation. Pelicans forage over relatively long distances (sometimes hundreds of kilometers; [14]), so the isotopic signatures in their feathers can be derived from multiple sources, which likely accounts for some of the variance observed. Sources of isotopic variance are often quite complex. Even in a highly simplified food web, Wunder et al. [57] detected high isotopic variance in four isotopes within a single moulting population of eared grebes (*Podiceps nigricollis*), which fed almost exclusively on brine shrimp (*Artemia franciscana*). Some of this variance appeared to be due to birds either not having reached isotopic equilibrium prior to moult or having moulted prior to arrival; however, even within feathers grown under isotopic equilibrium conditions, variance in isotopic signatures in this simplified food web were consistent with other studies from single locations with more complex food webs. Also, because breeding pelicans do not contain a single generation of feathers due to Staffelmauser in their flight feather moult, feathers from any given colony may include feathers grown in separate years [14,29]. Thus, if diet or precipitation patterns varied by year, temporal variance may have contributed to the high variation we observed. Finally, though flight feathers are grown on the breeding grounds [14, 29], our analysis may have included feathers lost and subsequently replaced during migration or on the wintering grounds or feathers from first-time breeders who moulted on the non-breeding grounds the previous year. This study highlights some of the challenges associated with using feather isotopes for tracking movement patterns, especially in a system with a complex dietary food web, Staffelmauser, and high movement capability.

Though the factors described above likely contribute, in part, to the overall isotopic variation we observed within colonies, a stronger contributing factor may be that some of the high variance in isotope values is due to year-to-year movement of some individuals among colonies. This explanation is consistent with the meta-population model often used to describe pelican population dynamics, as colonies frequently undergo large-scale fluctuations in population size, including local extinction events, suggesting frequent redistribution of individuals among breeding colonies [58–61]. The distance over which individuals redistribute after colony abandonment remains unclear; however, new colonies, such as at Akimiski Strait, NU have recently established hundreds of kilometers from previous range boundaries, suggesting the potential for long-distance breeding dispersal. If the high variance in isotope values observed here is due in part to long distance breeding dispersal, this could help explain the pattern of genetic panmixia observed in American white pelicans [12–13]. Low genetic differentiation is predicted in species with high dispersal capabilities [1], and our results are consistent with a high incidence of dispersal, which may be occurring frequently and at broad spatial scales, facilitating gene flow and, ultimately, panmixia.

## Supporting Information

S1 Table Sampling locations. Descriptive information for all sampling locations, including regional grouping, location information, sample size, and descriptive statistics for each isotope measured.
(DOCX)

S2 Table Cross-validation results. Results of a cross-validation resampling simulation from samples of known origin. Feathers were assigned to the site with the highest number of assignments out of the 10,000 simulations. During each simulation, samples were assigned to the site with the highest probability of origin.
(DOCX)
