# Supplementary material for: Linking Isotopes and Panmixia: High Within-Colony Variation in Feather δ2H, δ13C, and δ15N across the Range of the American White Pelican
Source: PLoS One. 2016 Mar 14;11(3):e0150810. doi: 10.1371/journal.pone.0150810 (PMC4790844; doi:10.1371/journal.pone.0150810)
Supplement: S1 Table — Descriptive information for all sampling locations, including regional grouping, location information, sample size, and descriptive statistics for each isotope measured. (DOCX) [file pone.0150810.s001.docx]

**S1 Table.** Descriptive information for all sampling locations, including regional grouping, location information, sample size, and descriptive statistics for each isotope measured.
