# Supplementary material for: Linking Isotopes and Panmixia: High Within-Colony Variation in Feather δ2H, δ13C, and δ15N across the Range of the American White Pelican
Source: PLoS One. 2016 Mar 14;11(3):e0150810. doi: 10.1371/journal.pone.0150810 (PMC4790844; doi:10.1371/journal.pone.0150810)
Supplement: S2 Table — Results of a cross-validation resampling simulation from samples of known origin. Feathers were assigned to the site with the highest number of assignments out of the 10,000 simulations. During each simulation, samples were assigned to the site with the highest probability of origin. (DOCX) [file pone.0150810.s002.docx]

**S2 Table.** Results of a cross-validation resampling simulation from samples of known origin. Feathers were assigned to the site with the highest number of assignments out of the 10,000 simulations. During each simulation, samples were assigned to the site with the highest probability of origin.

|  |  |  |  | **Assignment Confidence** | | | | | |
| --- | --- | --- | --- | --- | --- | --- | --- | --- | --- |
| **Individual** | **Sampling Location** | **Region Assigned** | **Assignment Correct?** | **100%** | **90%** | **80%** | **70%** | **50%** | **<50%** |
| ANIS-F-0001 | Anaho NWR, NV | Clear Lake, CA | N | N | N | N | Y | Y | N |
| ANIS-F-0002 | Anaho NWR, NV | Clear Lake, CA | N | N | N | N | Y | Y | N |
| ANIS-F-0003 | Anaho NWR, NV | Pipestone Rocks, MB | N | N | N | N | N | N | Y |
| ANIS-F-0004 | Anaho NWR, NV | Clear Lake, CA | N | N | N | Y | Y | Y | N |
| ANIS-F-0005 | Anaho NWR, NV | Bitter Lake, SD | N | N | Y | Y | Y | Y | N |
| ANIS-F-0006 | Anaho NWR, NV | Portage Lake, AB | N | N | N | N | N | Y | N |
| ANIS-F-0007 | Anaho NWR, NV | Clear Lake, CA | N | N | Y | Y | Y | Y | N |
| ANIS-F-0008 | Anaho NWR, NV | Blackfoot Reservoir, ID | N | N | N | Y | Y | Y | N |
| ANIS-F-0009 | Anaho NWR, NV | Clear Lake, CA | N | N | N | Y | Y | Y | N |
| ANIS-F-0010 | Anaho NWR, NV | Anaho NWR, NV | Y | N | N | N | N | Y | N |
| ANIS-F-0011 | Anaho NWR, NV | Blackfoot Reservoir, ID | N | N | N | N | N | Y | N |
| ANIS-F-0012 | Anaho NWR, NV | Mt. St. John, ON | N | N | N | N | N | Y | N |
| ANIS-F-0013 | Anaho NWR, NV | Anaho NWR, NV | Y | N | Y | Y | Y | Y | N |
| ANIS-F-0014 | Anaho NWR, NV | Anaho NWR, NV | Y | N | N | N | N | Y | N |
| ANIS-F-0015 | Anaho NWR, NV | Boles Island, ON | N | N | Y | Y | Y | Y | N |
| ANIS-F-0016 | Anaho NWR, NV | Clear Lake, CA | N | N | Y | Y | Y | Y | N |
| ANIS-F-0017 | Anaho NWR, NV | Clear Lake, CA | N | N | Y | Y | Y | Y | N |
| ANIS-F-0018 | Anaho NWR, NV | Clear Lake, CA | N | Y | Y | Y | Y | Y | N |
| ANIS-F-0019 | Anaho NWR, NV | Boles Island, ON | N | N | N | N | N | Y | N |
| BILA-F-0001 | Bitter Lake, SD | Bitter Lake, SD | Y | N | Y | Y | Y | Y | N |
| BILA-F-0002 | Bitter Lake, SD | Blackfoot Reservoir, ID | N | Y | Y | Y | Y | Y | N |
| BILA-F-0003 | Bitter Lake, SD | Lake of the Woods, ON | N | N | Y | Y | Y | Y | N |
| BILA-F-0004 | Bitter Lake, SD | Anaho NWR, NV | N | N | Y | Y | Y | Y | N |
| BILA-F-0005 | Bitter Lake, SD | Boles Island, ON | N | N | N | N | N | Y | N |
| BILA-F-0006 | Bitter Lake, SD | Anaho NWR, NV | N | N | N | N | Y | Y | N |
| BILA-F-0007 | Bitter Lake, SD | Chase Lake, ND | N | N | Y | Y | Y | Y | N |
| BILA-F-0008 | Bitter Lake, SD | Bitter Lake, SD | Y | N | Y | Y | Y | Y | N |
| BILA-F-0009 | Bitter Lake, SD | Pipestone Rocks, MB | N | N | N | N | N | Y | N |
| BILA-F-0010 | Bitter Lake, SD | Dore Lake, SK | N | N | N | N | N | Y | N |
| BILA-F-0011 | Bitter Lake, SD | Boles Island, ON | N | N | N | N | Y | Y | N |
| BILA-F-0012 | Bitter Lake, SD | Bitter Lake, SD | Y | N | N | Y | Y | Y | N |
| BILA-F-0013 | Bitter Lake, SD | Granite Island, ON | N | N | N | N | N | Y | N |
| BILA-F-0014 | Bitter Lake, SD | Boles Island, ON | N | N | N | N | N | Y | N |
| BILA-F-0015 | Bitter Lake, SD | Clear Lake, CA | N | N | N | N | N | Y | N |
| BRID-F-0024 | Blackfoot Reservoir, ID | Blackfoot Reservoir, ID | Y | N | Y | Y | Y | Y | N |
| BRID-F-0025 | Blackfoot Reservoir, ID | Padre Islands, TX | N | N | Y | Y | Y | Y | N |
| BRID-F-0026 | Blackfoot Reservoir, ID | Blackfoot Reservoir, ID | Y | N | Y | Y | Y | Y | N |
| BRID-F-0028 | Blackfoot Reservoir, ID | Blackfoot Reservoir, ID | Y | N | N | Y | Y | Y | N |
| BRID-F-0029 | Blackfoot Reservoir, ID | Clear Lake, CA | N | N | Y | Y | Y | Y | N |
| BRID-F-0030 | Blackfoot Reservoir, ID | Granite Island, ON | N | N | N | N | Y | Y | N |
| BRID-F-0031 | Blackfoot Reservoir, ID | Bitter Lake, SD | N | N | Y | Y | Y | Y | N |
| BRID-F-0032 | Blackfoot Reservoir, ID | Blackfoot Reservoir, ID | Y | N | Y | Y | Y | Y | N |
| BRID-F-0033 | Blackfoot Reservoir, ID | Blackfoot Reservoir, ID | Y | N | Y | Y | Y | Y | N |
| BOIS-F-0001 | Boles Island, ON | Clear Lake, CA | N | N | Y | Y | Y | Y | N |
| BOIS-F-0002 | Boles Island, ON | Boles Island, ON | Y | N | Y | Y | Y | Y | N |
| BOIS-F-0003 | Boles Island, ON | Ombabika Flats, ON | N | N | N | N | N | Y | N |
| BOIS-F-0004 | Boles Island, ON | Boles Island, ON | Y | N | N | N | N | Y | N |
| BOIS-F-0005 | Boles Island, ON | Mt. St. John, ON | N | N | Y | Y | Y | Y | N |
| BOIS-F-0006 | Boles Island, ON | Mt. St. John, ON | N | N | Y | Y | Y | Y | N |
| BOIS-F-0007 | Boles Island, ON | Boles Island, ON | Y | N | N | N | N | Y | N |
| BOIS-F-0008 | Boles Island, ON | Boles Island, ON | Y | Y | Y | Y | Y | Y | N |
| BOIS-F-0009 | Boles Island, ON | Boles Island, ON | Y | N | Y | Y | Y | Y | N |
| BOIS-F-0010 | Boles Island, ON | Mt. St. John, ON | N | Y | Y | Y | Y | Y | N |
| BOIS-F-0011 | Boles Island, ON | Boles Island, ON | Y | Y | Y | Y | Y | Y | N |
| BOIS-F-0012 | Boles Island, ON | Boles Island, ON | Y | N | Y | Y | Y | Y | N |
| BOIS-F-0013 | Boles Island, ON | Boles Island, ON | Y | Y | Y | Y | Y | Y | N |
| BOIS-F-0014 | Boles Island, ON | Boles Island, ON | Y | Y | Y | Y | Y | Y | N |
| BOIS-F-0015 | Boles Island, ON | Boles Island, ON | Y | Y | Y | Y | Y | Y | N |
| BOIS-F-0016 | Boles Island, ON | Boles Island, ON | Y | N | Y | Y | Y | Y | N |
| BOIS-F-0017 | Boles Island, ON | Boles Island, ON | Y | Y | Y | Y | Y | Y | N |
| BOIS-F-0018 | Boles Island, ON | Boles Island, ON | Y | Y | Y | Y | Y | Y | N |
| BOIS-F-0019 | Boles Island, ON | Boles Island, ON | Y | N | Y | Y | Y | Y | N |
| BOIS-F-0020 | Boles Island, ON | Boles Island, ON | Y | Y | Y | Y | Y | Y | N |
| CHAS-F-0001 | Chase Lake, ND | Mt. St. John, ON | N | N | Y | Y | Y | Y | N |
| CHAS-F-0002 | Chase Lake, ND | Chase Lake, ND | Y | N | N | N | N | Y | N |
| CHAS-F-0003 | Chase Lake, ND | Padre Islands, TX | N | N | Y | Y | Y | Y | N |
| CHAS-F-0004 | Chase Lake, ND | Blackfoot Reservoir, ID | N | N | Y | Y | Y | Y | N |
| CHAS-F-0005 | Chase Lake, ND | Bitter Lake, SD | N | N | Y | Y | Y | Y | N |
| CHAS-F-0006 | Chase Lake, ND | Ombabika Flats, ON | N | N | Y | Y | Y | Y | N |
| CLCA-F-0001 | Clear Lake, CA | Clear Lake, CA | Y | Y | Y | Y | Y | Y | N |
| CLCA-F-0002 | Clear Lake, CA | Clear Lake, CA | Y | N | Y | Y | Y | Y | N |
| CLCA-F-0003 | Clear Lake, CA | Portage Lake, AB | N | N | N | Y | Y | Y | N |
| CLCA-F-0004 | Clear Lake, CA | Clear Lake, CA | Y | Y | Y | Y | Y | Y | N |
| CLCA-F-0005 | Clear Lake, CA | Clear Lake, CA | Y | N | N | N | N | Y | N |
| CLCA-F-0006 | Clear Lake, CA | Portage Lake, AB | N | N | Y | Y | Y | Y | N |
| CLCA-F-0007 | Clear Lake, CA | Clear Lake, CA | Y | N | Y | Y | Y | Y | N |
| CLCA-F-0008 | Clear Lake, CA | Clear Lake, CA | Y | N | Y | Y | Y | Y | N |
| CLCA-F-0009 | Clear Lake, CA | Clear Lake, CA | Y | N | Y | Y | Y | Y | N |
| CLCA-F-0010 | Clear Lake, CA | Pipestone Rocks, MB | N | N | N | N | N | Y | N |
| CLCA-F-0011 | Clear Lake, CA | Utikuma Lake, AB | N | N | N | Y | Y | Y | N |
| CLCA-F-0012 | Clear Lake, CA | Clear Lake, CA | Y | N | Y | Y | Y | Y | N |
| CLCA-F-0013 | Clear Lake, CA | Clear Lake, CA | Y | Y | Y | Y | Y | Y | N |
| CLCA-F-0014 | Clear Lake, CA | Clear Lake, CA | Y | N | N | N | N | Y | N |
| CLCA-F-0015 | Clear Lake, CA | Anaho NWR, NV | N | N | Y | Y | Y | Y | N |
| DLSK-F-0002 | Dore Lake, SK | Chase Lake, ND | N | N | Y | Y | Y | Y | N |
| DLSK-F-0003 | Dore Lake, SK | Utikuma Lake, AB | N | N | N | N | Y | Y | N |
| DLSK-F-0004 | Dore Lake, SK | Mt. St. John, ON | N | N | N | N | Y | Y | N |
| DLSK-F-0006 | Dore Lake, SK | Last Mountain Lake, SK | N | N | N | N | Y | Y | N |
| DLSK-F-0007 | Dore Lake, SK | Pipestone Rocks, MB | N | N | Y | Y | Y | Y | N |
| DLSK-F-0008 | Dore Lake, SK | Dore Lake, SK | Y | N | N | N | N | Y | N |
| GRAN-F-0001 | Granite Island, ON | Granite Island, ON | Y | N | Y | Y | Y | Y | N |
| GRAN-F-0002 | Granite Island, ON | Dore Lake, SK | N | N | Y | Y | Y | Y | N |
| GRAN-F-0003 | Granite Island, ON | Blackfoot Reservoir, ID | N | N | Y | Y | Y | Y | N |
| GRAN-F-0004 | Granite Island, ON | Granite Island, ON | Y | Y | Y | Y | Y | Y | N |
| GRAN-F-0005 | Granite Island, ON | Ombabika Flats, ON | N | N | Y | Y | Y | Y | N |
| GRAN-F-0006 | Granite Island, ON | Granite Island, ON | Y | Y | Y | Y | Y | Y | N |
| GRAN-F-0007 | Granite Island, ON | Granite Island, ON | Y | N | Y | Y | Y | Y | N |
| GRAN-F-0008 | Granite Island, ON | Granite Island, ON | Y | N | Y | Y | Y | Y | N |
| LOTW-F-0001 | Lake of the Woods, ON | Mt. St. John, ON | N | N | Y | Y | Y | Y | N |
| LOTW-F-0002 | Lake of the Woods, ON | Padre Islands, TX | N | N | Y | Y | Y | Y | N |
| LOTW-F-0003 | Lake of the Woods, ON | Lake of the Woods, ON | Y | N | N | N | Y | Y | N |
| LOTW-F-0004 | Lake of the Woods, ON | Lake of the Woods, ON | Y | N | N | Y | Y | Y | N |
| LOTW-F-0005 | Lake of the Woods, ON | Pipestone Rocks, MB | N | N | Y | Y | Y | Y | N |
| LOTW-F-0006 | Lake of the Woods, ON | Lake of the Woods, ON | Y | N | N | N | N | N | Y |
| LOTW-F-0007 | Lake of the Woods, ON | Anaho NWR, NV | N | N | Y | Y | Y | Y | N |
| LOTW-F-0008 | Lake of the Woods, ON | Pipestone Rocks, MB | N | N | Y | Y | Y | Y | N |
| LOTW-F-0009 | Lake of the Woods, ON | Anaho NWR, NV | N | N | Y | Y | Y | Y | N |
| LOTW-F-0010 | Lake of the Woods, ON | Lake of the Woods, ON | Y | N | Y | Y | Y | Y | N |
| LML-F-0001 | Last Mountain Lake, SK | Portage Lake, AB | N | N | Y | Y | Y | Y | N |
| LML-F-0002 | Last Mountain Lake, SK | Last Mountain Lake, SK | Y | Y | Y | Y | Y | Y | N |
| LML-F-0003 | Last Mountain Lake, SK | Last Mountain Lake, SK | Y | Y | Y | Y | Y | Y | N |
| LML-F-0004 | Last Mountain Lake, SK | Last Mountain Lake, SK | Y | N | Y | Y | Y | Y | N |
| LML-F-0005 | Last Mountain Lake, SK | Last Mountain Lake, SK | Y | N | N | Y | Y | Y | N |
| LML-F-0006 | Last Mountain Lake, SK | Last Mountain Lake, SK | Y | Y | Y | Y | Y | Y | N |
| LML-F-0007 | Last Mountain Lake, SK | Last Mountain Lake, SK | Y | N | N | N | N | Y | N |
| LML-F-0008 | Last Mountain Lake, SK | Last Mountain Lake, SK | Y | N | N | N | Y | Y | N |
| LML-F-0009 | Last Mountain Lake, SK | Last Mountain Lake, SK | Y | N | Y | Y | Y | Y | N |
| LML-F-0010 | Last Mountain Lake, SK | Last Mountain Lake, SK | Y | N | Y | Y | Y | Y | N |
| MLMN-F-0001 | Marsh Lake, MN | Bitter Lake, SD | N | N | N | N | Y | Y | N |
| MLMN-F-0002 | Marsh Lake, MN | Anaho NWR, NV | N | N | Y | Y | Y | Y | N |
| MLMN-F-0003 | Marsh Lake, MN | Granite Island, ON | N | N | N | N | N | N | Y |
| MLMN-F-0004 | Marsh Lake, MN | Mt. St. John, ON | N | N | Y | Y | Y | Y | N |
| MLMN-F-0005 | Marsh Lake, MN | Mt. St. John, ON | N | N | N | N | N | Y | N |
| MLMN-F-0006 | Marsh Lake, MN | Blackfoot Reservoir, ID | N | N | N | N | N | Y | N |
| MLMN-F-0007 | Marsh Lake, MN | Utikuma Lake, AB | N | N | N | N | N | Y | N |
| MLMN-F-0008 | Marsh Lake, MN | Granite Island, ON | N | N | N | N | N | Y | N |
| MLMN-F-0009 | Marsh Lake, MN | Boles Island, ON | N | N | Y | Y | Y | Y | N |
| MLMN-F-0010 | Marsh Lake, MN | Granite Island, ON | N | N | N | N | N | Y | N |
| MLMN-F-0011 | Marsh Lake, MN | Blackfoot Reservoir, ID | N | N | N | N | N | N | Y |
| MLMN-F-0012 | Marsh Lake, MN | Anaho NWR, NV | N | N | N | N | N | N | Y |
| MLMN-F-0013 | Marsh Lake, MN | Blackfoot Reservoir, ID | N | N | Y | Y | Y | Y | N |
| MLMN-F-0014 | Marsh Lake, MN | Bitter Lake, SD | N | N | N | Y | Y | Y | N |
| MLMN-F-0015 | Marsh Lake, MN | Bitter Lake, SD | N | N | N | N | N | Y | N |
| MTSJ-F-0001 | Mt. St. John, ON | Boles Island, ON | N | N | Y | Y | Y | Y | N |
| MTSJ-F-0002 | Mt. St. John, ON | Boles Island, ON | N | N | Y | Y | Y | Y | N |
| MTSJ-F-0003 | Mt. St. John, ON | Boles Island, ON | N | Y | Y | Y | Y | Y | N |
| MTSJ-F-0004 | Mt. St. John, ON | Mt. St. John, ON | Y | N | Y | Y | Y | Y | N |
| MTSJ-F-0005 | Mt. St. John, ON | Blackfoot Reservoir, ID | N | N | Y | Y | Y | Y | N |
| MTSJ-F-0006 | Mt. St. John, ON | Mt. St. John, ON | Y | N | Y | Y | Y | Y | N |
| MTSJ-F-0007 | Mt. St. John, ON | Boles Island, ON | N | N | N | N | N | Y | N |
| MTSJ-F-0008 | Mt. St. John, ON | Mt. St. John, ON | Y | Y | Y | Y | Y | Y | N |
| MTSJ-F-0009 | Mt. St. John, ON | Granite Island, ON | N | N | N | N | Y | Y | N |
| MTSJ-F-0010 | Mt. St. John, ON | Mt. St. John, ON | Y | N | Y | Y | Y | Y | N |
| OMBA-F-0001 | Ombabika Flats, ON | Pipestone Rocks, MB | N | N | N | N | N | N | Y |
| OMBA-F-0002 | Ombabika Flats, ON | Pipestone Rocks, MB | N | N | N | N | Y | Y | N |
| OMBA-F-0003 | Ombabika Flats, ON | Mt. St. John, ON | N | N | N | N | Y | Y | N |
| OMBA-F-0004 | Ombabika Flats, ON | Bitter Lake, SD | N | N | N | N | Y | Y | N |
| OMBA-F-0005 | Ombabika Flats, ON | Mt. St. John, ON | N | N | N | N | N | Y | N |
| OMBA-F-0006 | Ombabika Flats, ON | Granite Island, ON | N | N | N | Y | Y | Y | N |
| OMBA-F-0007 | Ombabika Flats, ON | Pipestone Rocks, MB | N | N | N | Y | Y | Y | N |
| OMBA-F-0008 | Ombabika Flats, ON | Anaho NWR, NV | N | N | Y | Y | Y | Y | N |
| OMBA-F-0009 | Ombabika Flats, ON | Ombabika Flats, ON | Y | N | N | N | N | Y | N |
| OMBA-F-0010 | Ombabika Flats, ON | Granite Island, ON | N | N | Y | Y | Y | Y | N |
| TEXS-F-0001 | Padre Islands, TX | Padre Islands, TX | Y | N | Y | Y | Y | Y | N |
| TEXS-F-0002 | Padre Islands, TX | Padre Islands, TX | Y | N | Y | Y | Y | Y | N |
| TEXS-F-0003 | Padre Islands, TX | Padre Islands, TX | Y | N | Y | Y | Y | Y | N |
| TEXS-F-0004 | Padre Islands, TX | Padre Islands, TX | Y | Y | Y | Y | Y | Y | N |
| TEXS-F-0005 | Padre Islands, TX | Padre Islands, TX | Y | Y | Y | Y | Y | Y | N |
| TEXS-F-0006 | Padre Islands, TX | Padre Islands, TX | Y | Y | Y | Y | Y | Y | N |
| TEXS-F-0007 | Padre Islands, TX | Padre Islands, TX | Y | N | Y | Y | Y | Y | N |
| TEXS-F-0008 | Padre Islands, TX | Padre Islands, TX | Y | N | Y | Y | Y | Y | N |
| TEXS-F-0009 | Padre Islands, TX | Padre Islands, TX | Y | N | Y | Y | Y | Y | N |
| TEXS-F-0010 | Padre Islands, TX | Pipestone Rocks, MB | N | N | N | N | N | Y | N |
| PIRO-F-0001 | Pipestone Rocks, MB | Pipestone Rocks, MB | Y | N | Y | Y | Y | Y | N |
| PIRO-F-0002 | Pipestone Rocks, MB | Pipestone Rocks, MB | Y | N | Y | Y | Y | Y | N |
| PIRO-F-0003 | Pipestone Rocks, MB | Granite Island, ON | N | N | Y | Y | Y | Y | N |
| PIRO-F-0004 | Pipestone Rocks, MB | Pipestone Rocks, MB | Y | N | N | Y | Y | Y | N |
| PIRO-F-0005 | Pipestone Rocks, MB | Anaho NWR, NV | N | N | N | Y | Y | Y | N |
| PIRO-F-0006 | Pipestone Rocks, MB | Pipestone Rocks, MB | Y | N | N | N | N | Y | N |
| PIRO-F-0007 | Pipestone Rocks, MB | Anaho NWR, NV | N | N | N | N | Y | Y | N |
| PIRO-F-0008 | Pipestone Rocks, MB | Chase Lake, ND | N | N | N | N | Y | Y | N |
| PIRO-F-0009 | Pipestone Rocks, MB | Pipestone Rocks, MB | Y | N | N | N | N | Y | N |
| PIRO-F-0010 | Pipestone Rocks, MB | Blackfoot Reservoir, ID | N | N | Y | Y | Y | Y | N |
| PLAB-F-0001 | Portage Lake, AB | Last Mountain Lake, SK | N | N | Y | Y | Y | Y | N |
| PLAB-F-0002 | Portage Lake, AB | Portage Lake, AB | N | N | N | Y | Y | Y | N |
| PLAB-F-0003 | Portage Lake, AB | Portage Lake, AB | N | N | N | Y | Y | Y | N |
| PLAB-F-0004 | Portage Lake, AB | Portage Lake, AB | N | N | Y | Y | Y | Y | N |
| PLAB-F-0005 | Portage Lake, AB | Portage Lake, AB | N | N | Y | Y | Y | Y | N |
| PLAB-F-0006 | Portage Lake, AB | Portage Lake, AB | N | N | Y | Y | Y | Y | N |
| PLAB-F-0007 | Portage Lake, AB | Portage Lake, AB | N | N | N | N | N | Y | N |
| PLAB-F-0008 | Portage Lake, AB | Last Mountain Lake, SK | N | N | N | N | N | Y | N |
| PLAB-F-0009 | Portage Lake, AB | Last Mountain Lake, SK | N | N | Y | Y | Y | Y | N |
| PLAB-F-0010 | Portage Lake, AB | Mt. St. John, ON | N | N | Y | Y | Y | Y | N |
| PLAB-F-0011 | Portage Lake, AB | Pipestone Rocks, MB | N | N | N | N | N | Y | N |
| PLAB-F-0012 | Portage Lake, AB | Last Mountain Lake, SK | N | N | Y | Y | Y | Y | N |
| PLAB-F-0013 | Portage Lake, AB | Last Mountain Lake, SK | N | N | Y | Y | Y | Y | N |
| PLAB-F-0014 | Portage Lake, AB | Mt. St. John, ON | N | N | N | N | N | Y | N |
| PLAB-F-0015 | Portage Lake, AB | Last Mountain Lake, SK | N | N | N | Y | Y | Y | N |
| ULAB-F-0001 | Utikuma Lake, AB | Lake of the Woods, ON | N | N | N | Y | Y | Y | N |
| ULAB-F-0002 | Utikuma Lake, AB | Granite Island, ON | N | N | N | N | Y | Y | N |
| ULAB-F-0003 | Utikuma Lake, AB | Mt. St. John, ON | N | N | N | Y | Y | Y | N |
| ULAB-F-0004 | Utikuma Lake, AB | Utikuma Lake, AB | Y | N | N | Y | Y | Y | N |
| ULAB-F-0005 | Utikuma Lake, AB | Clear Lake, CA | N | N | Y | Y | Y | Y | N |
| ULAB-F-0006 | Utikuma Lake, AB | Blackfoot Reservoir, ID | N | N | N | Y | Y | Y | N |
| ULAB-F-0007 | Utikuma Lake, AB | Clear Lake, CA | N | N | Y | Y | Y | Y | N |
| ULAB-F-0008 | Utikuma Lake, AB | Utikuma Lake, AB | Y | N | Y | Y | Y | Y | N |
| ULAB-F-0009 | Utikuma Lake, AB | Pipestone Rocks, MB | N | N | N | N | Y | Y | N |
